# Circular RNA circSEMA5A promotes bladder cancer progression by upregulating ENO1 and SEMA5A expression

**DOI:** 10.18632/aging.103971

**Published:** 2020-11-07

**Authors:** Lei Wang, Haoran Li, Qingdong Qiao, Yukun Ge, Ling Ma, Qiang Wang

**Affiliations:** 1 Department of Urology, Xinxiang Central Hospital, Xinxiang, Henan, China; 2 School of Basic Medical Sciences, Xinxiang Medical University, Xinxiang, Henan, China; 3 Department of Urology, Beijing Friendship Hospital, Capital Medical University, Beijing, China

**Keywords:** bladder cancer, circular RNA, miR-330-5p, Enolase 1, SEMA5A

## Abstract

Bladder cancer (BC) is one of the most commonly diagnosed urologic carcinomas, with high recurrence and death rates. Circular RNAs (circRNAs) are a class of noncoding RNAs which are anomalously expressed in cancers and involved in the progression of cancers. In this study, we found that circSEMA5A was upregulated in BC tissues and cell lines. The overexpressed circSEMA5A was correlated with malignant characteristics of BC. In vitro data indicated that circSEMA5A promoted proliferation, suppressed apoptosis, facilitated migration, accelerated invasion, enhanced angiogenesis and promotes glycolysis of BC. Mechanistically, circSEMA5A served as a miRNA sponge for miR-330-5p to upregulates Enolase 1 (ENO1) expression and facilitated the activation of Akt and β-catenin signaling pathways. Then, we showed that circSEMA5A exerted its biological functions partially via miR-330-5p/ENO1 signaling. Moreover, circSEMA5A raised SEMA5A expression by recruiting EIF4A3 to enhance the mRNA stability of SEMA5A, and thereby accelerated BC angiogenesis. To sum up, circSEMA5A is upregulated in BC and facilitates BC progression by mediating miR-330-5p/ENO1 signaling and upregulating SEMA5A expression.

## INTRODUCTION

Bladder cancer (BC) has become one of the most commonly diagnosed urologic carcinomas, with high recurrence and death rates [1, 2]. Though multiple therapeutic approaches have been used for BC treatment, the prognosis of many patients still remains very poor, especially the patients with muscle-invasive bladder cancer (MIBC) [3, 4]. Multiple mechanisms promote the malignant process of BC which are still largely unclear, bringing great impediments to the diagnosis and treatment of BC. Thus, it is of great significance to have a better understanding of molecular mechanisms that cause BC.

Circular RNAs (circRNAs) are a novel class of noncoding RNA molecules that formed by a covalently closed loop structures without a 5’cap and a 3’poly A tail [5]. Increasing evidence indicates that circRNAs are anomalously expressed in various cancers and involved in the progression of these cancers [6, 7]. A common mechanism by which circRNAs exert their functions is to serve as microRNA (miRNA) sponges to restrain the inhibition effect of miRNAs on their target genes [8, 9]. In BC, there are also some circRNAs abnormally expressed, and participate in the malignant process of BC through this mechanism. For example, circPTPRA is down-regulated in BC and acts as a tumor suppressor in BC by sponging miR-636 and upregulating KLF9 [10]. Hsa_circ_0001361 is highly expressed in BC and promotes bladder cancer invasion and metastasis through miR-491-5p/MMP9 axis [11]. CircNR3C1 is downregulated in BC and inhibits proliferation of bladder cancer cells by sponging miR-27a-3p and downregulating cyclin D1 expression [12]. Hence, circRNAs may play important roles in BC progression. However, there are only several circRNAs have been investigated in BC and the functions of most circRNAs in BC remain unclear.

In this study, we studied hsa_circ_0071820 which originated from exons 3 and 4 of the SEMA5A gene, termed circSEMA5A. CircSEMA5A was firstly identified to be upregulated in BC through circRNA sequencing by Li et al [13] and we further confirmed it by qRT-PCR. Subsequently, functional studies discovered that circSEMA5A promoted malignant processes of BC cells in vitro.

Mechanistically, circSEMA5A could not only upregulate ENO1 by sponging miR-330-5p and then activated AKT, β-catenin and AMPK signaling pathways, but also raise SEMA5A expression by recruiting EIF4A3 to enhance the mRNA stability of SEMA5A. Our study revealed the role of the circSEMA5A in BC which might provide potential therapeutic targets for BC.

## RESULTS

### circSEMA5A is upregulated in BC

**Figure 1:**
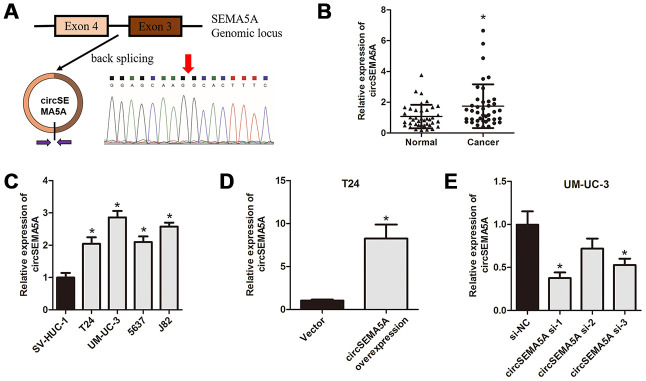
**circSEMA5A is upregulated in BC.** (**A**) Diagram showed the circSEMA5A derived from exons 3 and 4 of SEMA5A gene. The back-splicing junction site of circSEMA5A was confirmed by PCR amplification and followed by Sanger sequencing. (**B**) The relative expression of circSEMA5A was detected by qRT-PCR in BC tissues and paired normal bladder tissues. (**C**) The relative expression of circSEMA5A was detected by qRT-PCR in BC cell lines and a normal human uroepithelial cell line. (**D** and **E**) The transfection efficiencies were verified by qRT-PCR when T24 cells were transfected with circSEMA5A overexpression plasmids and UM-UC-3 cells were transfected with circSEMA5A siRNAs. Data are presented as mean ± SD. *P < 0.05.

First, we used qRT-PCR to detect the expressions of circSEMA5A in BC tissues and cell lines. CircSEMA5A is derived from exons 3 and 4 of the SEMA5A gene, and the junction site was verified by Sanger sequencing using qRT-PCR products (Figure 1A). The results showed that circSEMA5A expression was overexpressed in both BC tissues and BC cell lines (Figure 1B and 1C). Next, we analyzed the clinical value of circSEMA5A expression in BC. Through correlation analysis with clinicopathological factors, we found circSEMA5A expression was associated with tumor stage and tumor size (Table 1). All these results showed that circSEMA5A was upregulated in BC and correlated with malignant characteristics of BC, which indicated an oncogenic role of circSEMA5A in BC. To further investigate the functional roles of circSEMA5A in BC cells, circSEMA5A overexpression plasmids and siRNAs were transfected into T24 and UM-UC-3 cells, respectively. The transfection efficiencies were then verified by qRT-PCR (Figure 1D and 1E).

**Table 1:** Correlation between circSEMA5A expression and clinicopathological characteristics of bladder cancer patients.

**Characteristics**	**Case**	**circSEMA5A expression**		***P* value**
		**Low**	**High**	
Age(years)				
< 65	24	11	13	
≥ 65	16	9	7	0.518
Gender				
Male	34	18	16	
Female	6	2	4	0.376
Tumor stage				
Ta-T1	14	10	4	
T2-T4	26	10	16	0.047
Lymph nodes status				
Negative	33	18	15	
Positive	7	2	5	0.212
Histological grade				
Low	10	7	3	
High	30	13	17	0.784
Tumor size (cm)				
< 3	25	16	9	
≥ 3	15	4	11	0.022
Multifocality				
Unifocal	32	18	14	
Multifocal	8	2	6	0.114

*P<0.05 was considered statistically significant (Chi-square test).

### CircSEMA5A accelerates the oncogenic process of BC

**Figure 2:**
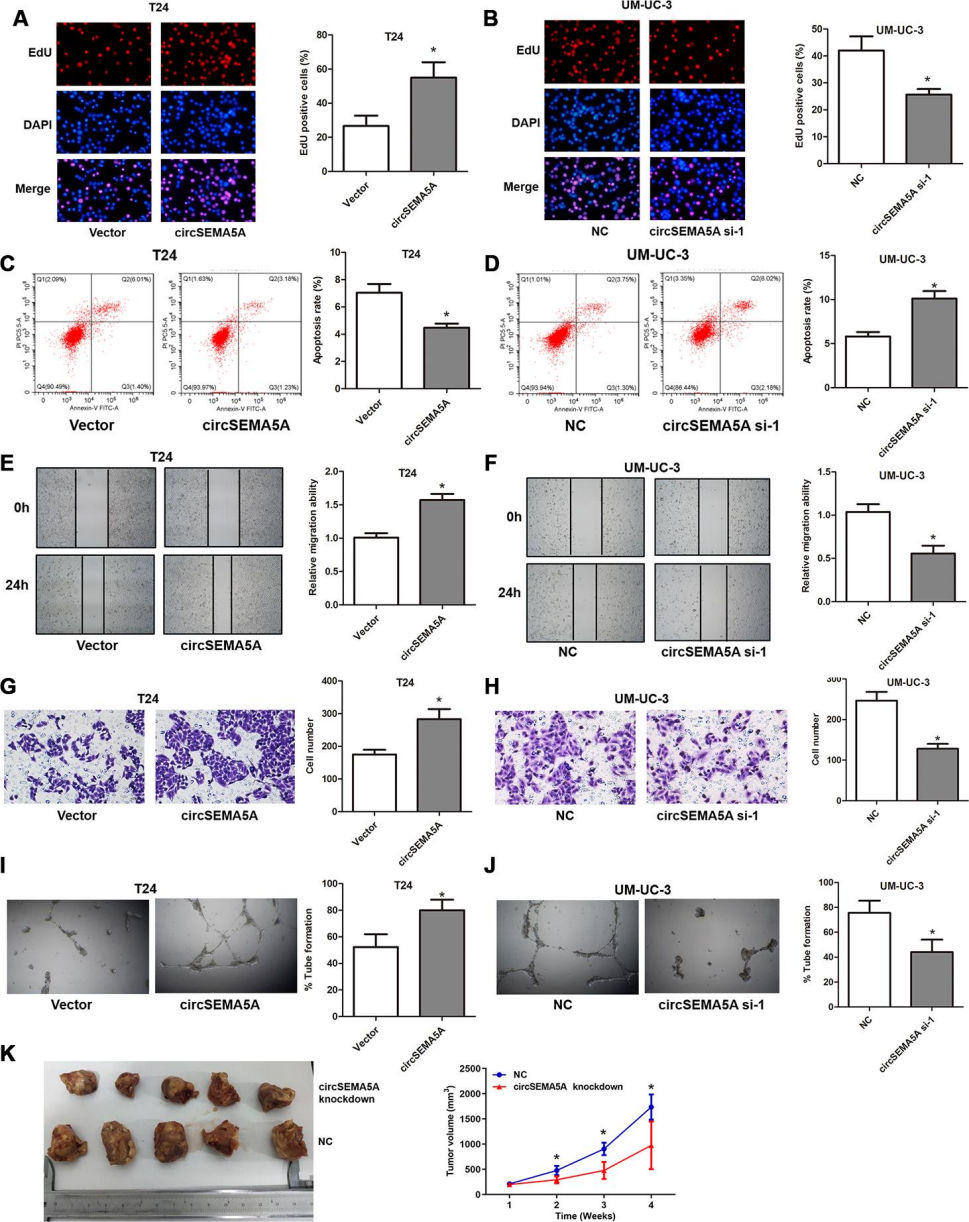
**CircSEMA5A accelerates the oncogenic process of BC cells.** (**A**–**J**) T24 cells were transfected with circSEMA5A overexpression plasmids and UM-UC-3 cells were transfected with circSEMA5A siRNAs. (**A** and **B**) Cell proliferation was measured by EdU assays; (**C** and **D**) cell apoptosis was detected by flow cytometry; (**E** and **F**) cell migration was assessed using wound-healing assays; (**G** and **H**) cell invasion was evaluated by transwell assays; (**I** and **J**) angiogenesis capability was assessed by tube formation assay. (**K**) Xenograft assay assessing the effect of circSEMA5A knockdown on tumor growth in vivo. Data are presented as mean ± SD. *P < 0.05.

Subsequently, we evaluated the potential biological effect of circSEMA5A on BC progression in vitro and in vivo. We observed that overexpressing circSEMA5A in T24 cells significantly promoted proliferation ability (Figure 2A), suppressed apoptosis (Figure 2C), facilitated migration capability (Figure 2E), accelerated invasion ability (Figure 2G) and promoted angiogenesis (Figure 2I) in vitro. Accordingly, silencing circSEMA5A in UM-UC-3 cells significantly restrained proliferation ability (Figure 2B), promoted apoptosis (Figure 2D), suppressed migration capability (Figure 2F), repressed invasion ability (Figure 2H) and restrained angiogenesis (Figure 2J) in vitro. Moreover, silencing circSEMA5A also suppressed tumor growth in vivo (Figure 2K). We concluded that circSEMA5A promoted the oncogenic process of BC.

### CircSEMA5A promotes glycolysis of BC cells

**Figure 3:**
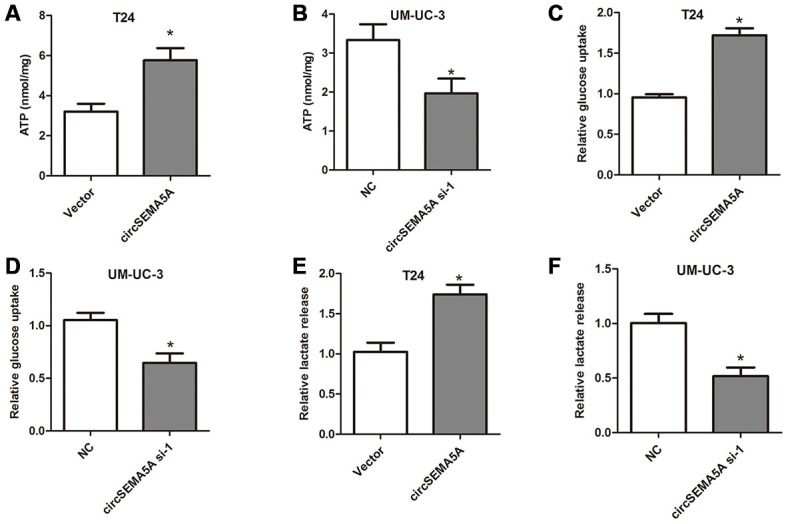
**CircSEMA5A promotes glycolysis of BC cells.** (**A**–**B**) T24 cells were transfected with circSEMA5A overexpression plasmids and UM-UC-3 cells were transfected with circSEMA5A siRNAs. The level of ATP (**A** and **B**), relative glucose uptake (**C** and **D**) and lactate production (**E** and **F**) were subsequently measured. Data are presented as mean ± SD. *P < 0.05.

Moreover, we also investigated the effect of circSEMA5A on BC cells glycolysis. When T24 cells were transfected with circSEMA5A overexpression plasmids, the level of ATP, relative glucose uptake and lactate production increased (Figure 3A, 3C and 3E). Accordingly, when UM-UC-3 cells were transfected with circSEMA5A siRNAs, the level of ATP, relative glucose uptake and lactate production decreased (Figure 3B, 3D and 3F). These data demonstrated that circSEMA5A promoted glycolysis of BC cells.

### CircSEMA5A serves as a miRNA sponge for miR-330-5p to upregulates ENO1 expression

**Figure 4:**
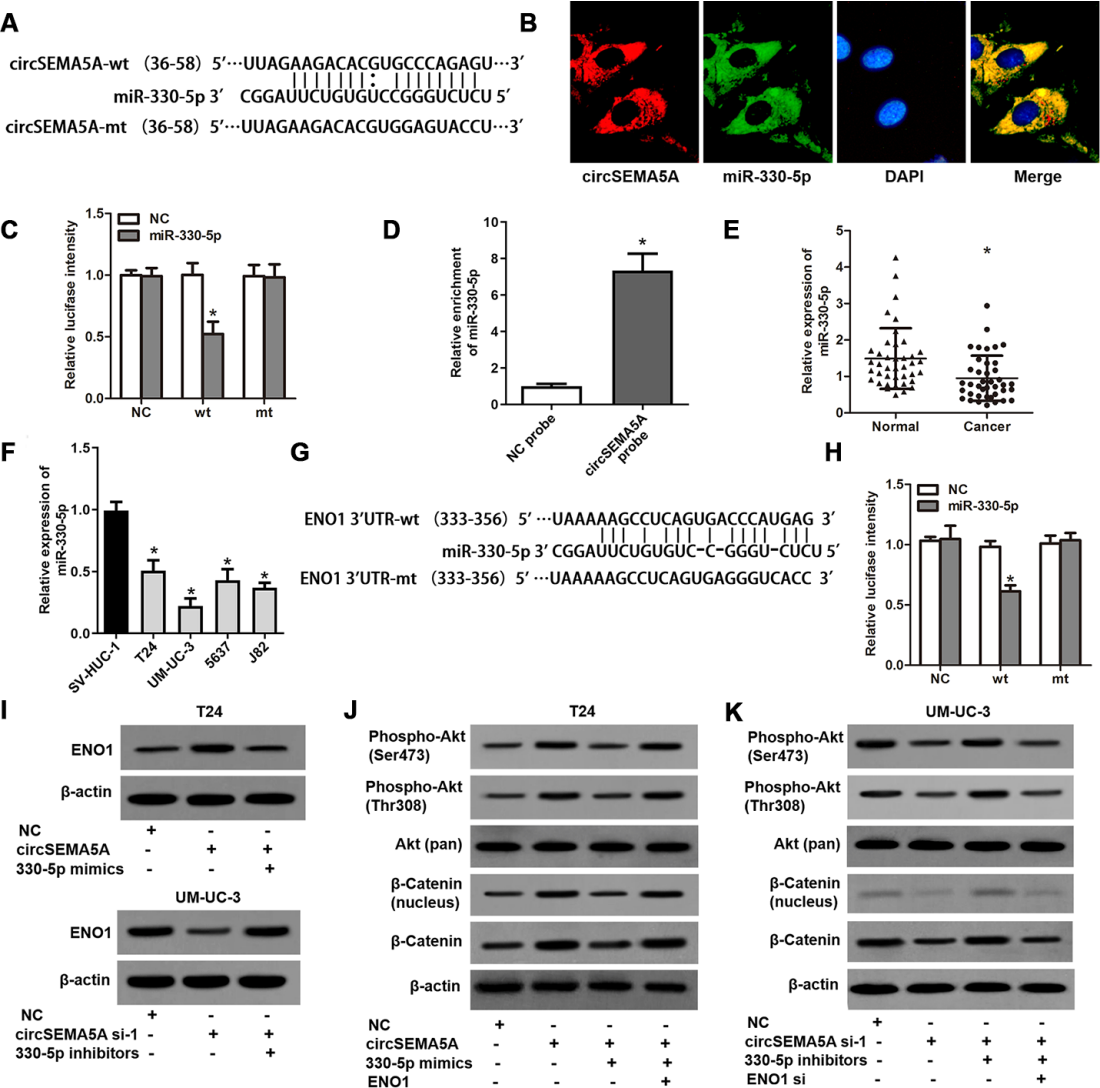
**CircSEMA5A serves as a miRNA sponge for miR-330-5p to upregulates ENO1 expression.** (**A**) Diagram showed the potential binding site of miR-330-5p in circSEMA5A sequence. (**B**) Co-localization of circSEMA5A and miR-330-5p by FISH assays. (**C**) Luciferase reporter assay verified the predicted binding site between miR-330-5p and circSEMA5A. (**D**) The level of miR-330-5p pulled-down by circSEMA5A probe was analyzed by qRT-PCR. (**E**) The relative expression of miR-330-5p was detected by qRT-PCR in BC tissues and paired normal bladder tissues. (**F**) The relative expression of miR-330-5p was detected by qRT-PCR in BC cell lines and a normal human uroepithelial cell line. (**G**) Diagram showed the potential binding site of miR-330-5p in ENO1 3’UTR. (**H**) Luciferase reporter assay verified the predicted binding site between miR-330-5p and ENO1 3’UTR. (**I**) T24 cells were transfected with circSEMA5A overexpression plasmids and miR-330-5p mimics and UM-UC-3 cells were transfected with circSEMA5A siRNAs and miR-330-5p inhibitors, and ENO1 expression was detected by western blot assay. (**J** and **K**) Activations of Akt and β-catenin signaling pathways were evaluated by western blot assay. Data are presented as mean ± SD. *P < 0.05.

Through Circular RNA Interactome database, we found many miRNAs which might interact with circSEMA5A. Among these miRNAs, miR-330-5p got the highest context+ score percentile. So we chose miR-330-5p to verify the binding site (Figure 4A). FISH assays indicated that both circSEMA5A and miR-330-5p mostly localized in the cytoplasm (Figure 4B). Luciferase reporter assay displayed that transfection of miR-330-5p could only reduce the intensity of luciferase reporter carrying the wild-type circSEMA5A sequence (Figure 4C). Additionally, RNA pull-down assay confirmed the binding of miR-330-5p to circSEMA5A (Figure 4D). We also found that miR-330-5p was significantly downregulated in BC tissues and cell lines (Figure 4E and 4F). Through microRNA.org, we identified ENO1 was a direct target of miR-330-5p (Figure 4G). This interaction was also verified by luciferase reporter assays (Figure 4H). As a result, we revealed that circSEMA5A raised ENO1 expression via regulating miR-330-5p (Figure 4I). Additionally, the Akt and β-catenin signaling pathways were found to be activated by circSEMA5A/miR-330-5p/ENO1 axis (Figure 4J and 4K).

### CircSEMA5A facilitates the oncogenic process and glycolysis of BC cells via miR-330-5p/ENO1

**Figure 5:**
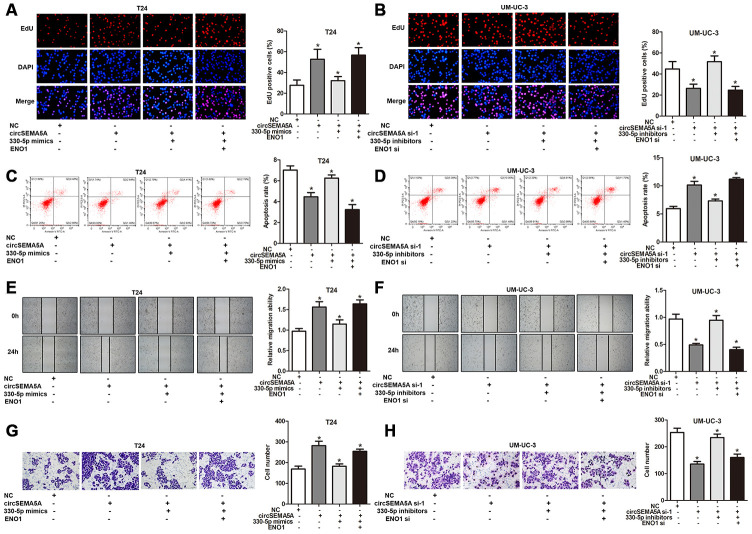
**CircSEMA5A facilitates the oncogenic process of BC cells via miR-330-5p/ENO1.** (**A**–**H**) T24 cells were transfected with circSEMA5A overexpression plasmids, miR-330-5p mimics and ENO1 overexpression plasmids; UM-UC-3 cells were transfected with circSEMA5A siRNAs, miR-330-5p inhibitors and ENO1 siRNAs. (**A** and **B**) Cell proliferation was measured by EdU assays; (**C** and **D**) cell apoptosis was detected by flow cytometry; (**E** and **F**) cell migration was assessed using wound-healing assays; (**G** and **H**) cell invasion was evaluated transwell assays. Data are presented as mean ± SD. *P < 0.05.

**Figure 6:**
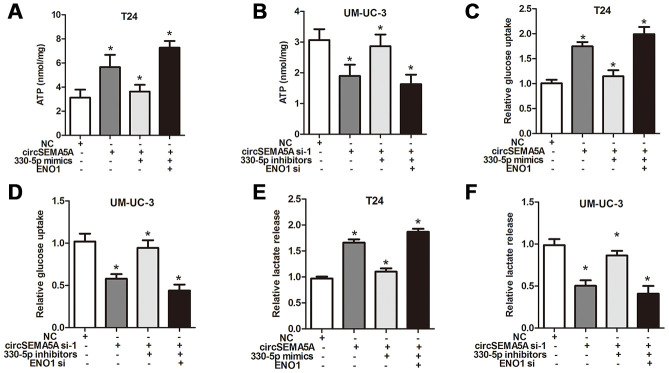
**CircSEMA5A facilitates the glycolysis of BC cells via miR-330-5p/ENO1.** (**A**–**F**) T24 cells were transfected with circSEMA5A overexpression plasmids, miR-330-5p mimics and ENO1 overexpression plasmids; UM-UC-3 cells were transfected with circSEMA5A siRNAs, miR-330-5p inhibitors and ENO1 siRNAs. The level of ATP (**A** and **B**), relative glucose uptake (**C** and **D**) and lactate production (**E** and **F**) were subsequently measured. Data are presented as mean ± SD. *P < 0.05.

As mentioned above, we proved that circSEMA5A could accelerated the oncogenic process of BC cells and circSEMA5A could facilitate ENO1 expression via miR-330-5p. Consequently, we considered that circSEMA5A might exert its biological effects via modulating miR-330-5p/ENO1 signaling. Then, T24 cells were transfected with circSEMA5A overexpression plasmids, miR-330-5p mimics and ENO1 overexpression plasmids; UM-UC-3 cells were transfected with circSEMA5A siRNAs, miR-330-5p inhibitors and ENO1 siRNAs. Subsequently, EdU assays (Figure 5A and 5B), cell apoptosis assays (Figure 5C and 5D), wound-healing assays (Figure 5E and 5F) and transwell assays (Figure 5G and 5H) displayed the modulation of the circSEMA5A/miR-330-5p/ENO1 signaling on cell proliferation, apoptosis, migration and invasion. At the same time, we tested the modulation of the circSEMA5A/miR-330-5p/ENO1 signaling on glycolysis of BC cells (Figure 6A–6F). Taken together, the results demonstrated that circSEMA5A facilitates these oncogenic process and glycolysis of BC cells via miR-330-5p/ENO1 signaling.

### CircSEMA5A accelerates BC angiogenesis through upregulating SEMA5A

**Figure 7:**
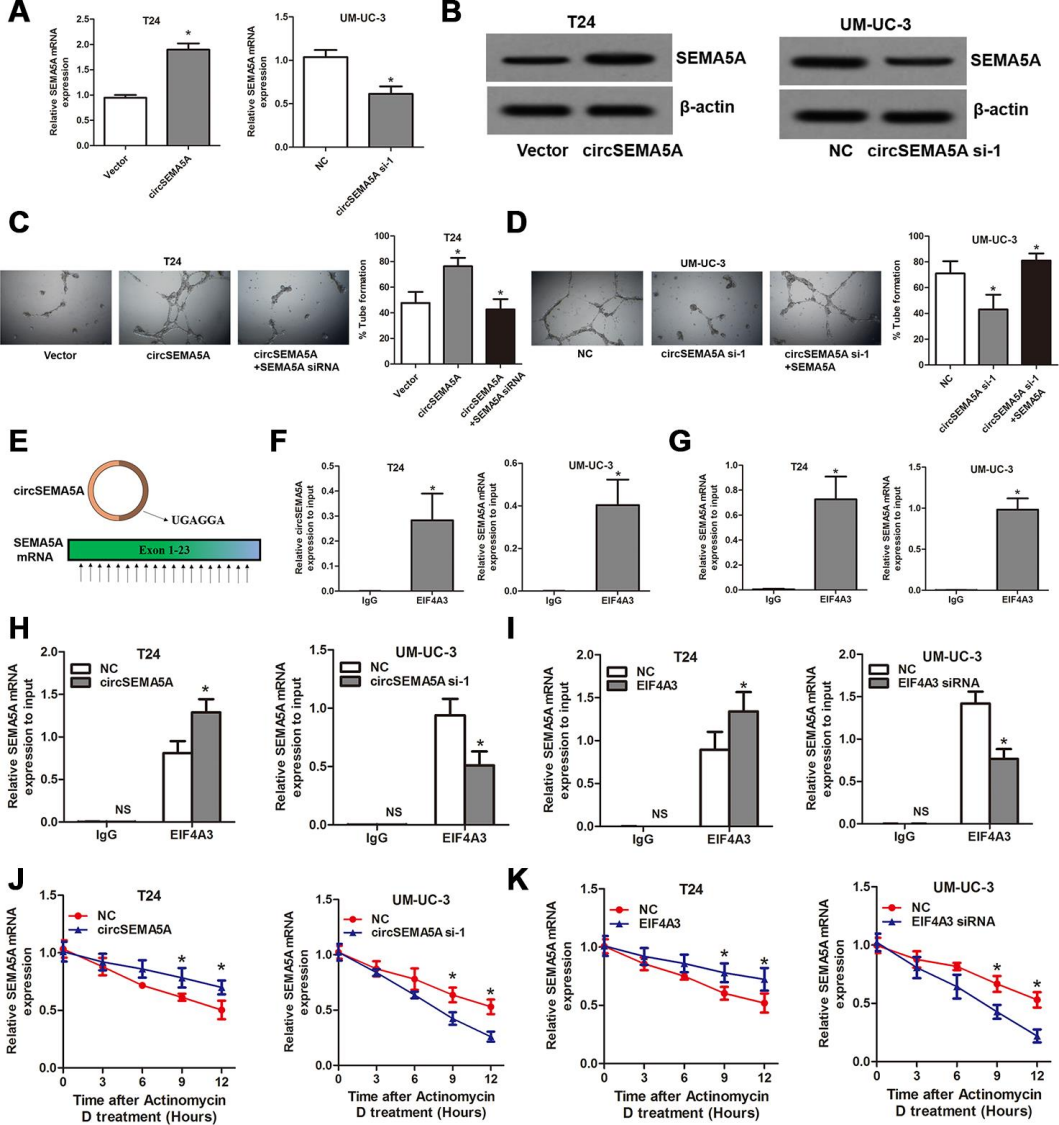
**CircSEMA5A accelerates BC angiogenesis through upregulating SEMA5A.** (**A** and **B**) qRT-PCR and western blot assays detecting SEMA5A expressions after T24 cells were transfected with circSEMA5A overexpression plasmids and UM-UC-3 cells were transfected with circSEMA5A siRNAs. (**C** and **D**) Angiogenesis capability was assessed by tube formation assay after T24 cells were transfected with circSEMA5A overexpression plasmids together with SEMA5A siRNAs and UM-UC-3 cells were transfected with circSEMA5A siRNAs together with SEMA5A overexpression plasmids. (**E**) Binding motif of EIF4A3 in circSEMA5A and SEMA5A mRNA. (**F** and **G**) RIP validated the interactions of EIF4A3 with circSEMA5A and SEMA5A mRNA. (**H** and **I**) Cells were transfected with circSEMA5A or EIF4A3 overexpression plasmids or siRNAs, the enrichments of SEMA5A mRNA in anti-EIF4A3 precipitates were analyzed by qRT-PCR and compared with NC group. (**J** and **K**) The influence of circSEMA5A or EIF4A3 on the stability of SEMA5A mRNA was assessed by RNA stability analysis. Data are presented as mean ± SD. NS, no significance; *P < 0.05.

We noticed that ENO1 didn’t possess the biological function of promoting angiogenesis, so, how did circSEMA5A affect angiogenesis? Here, we found that circSEMA5A could upregulate the mRNA and protein levels of SEMA5A (Figure 7A and 7B). SEMA5A was reported to be a transmembrane protein which could be both transmembrane and secreted and play critical roles in angiogenesis [14]. We then demonstrated that circSEMA5A affecting BC angiogenesis through regulating SEMA5A (Figure 7C and 7D). Subsequently, we investigated the potential mechanism of circSEMA5A in regulating SEMA5A. Through starBase data base, we identified a RNA binding protein EIF4A3 which could bind to circSEMA5A and SEMA5A. The exon 3 region of circSEMA5A contained a EIF4A3 binding motif sequence UGAGGA and there are many EIF4A3 binding sites in SEMA5A mRNA (Figure 7E). Then we performed RIP assays using EIF4A3 antibody to validate these interactions. The results suggested that both circSEMA5A and SEMA5A mRNA could be co-precipitated by EIF4A3 (Figure 7F and 7G). Moreover, the enrichments of SEMA5A mRNA in anti-EIF4A3 precipitates were augmented and diminished owing to circSEMA5A or EIF4A3 overexpression and silencing, respectively (Figure 7H and 7I). More importantly, circSEMA5A or EIF4A3 overexpression and silencing also influenced the stability of SEMA5A mRNA (Figure 7J and 7K). In summary, these results demonstrated that circSEMA5A raised SEMA5A expression by recruiting EIF4A3 to enhance the mRNA stability of SEMA5A, and thereby accelerated BC angiogenesis.

## DISCUSSION

CircRNAs are abundant, conserved, stable in cells and abnormally expressed in human diseases [15]. Increasing evidences indicate that circRNAs play important roles in the occurrence and development of various cancers [16, 17]. As for BC, more and more circRNAs have been identified to be deregulated by means of bioinformatics analysis and high-throughput sequencing [13, 18]. Despite all this, the functions and the mechanism of these circRNAs in BC remain largely unclear, and still need to be further investigated. Although circSEMA5A were found to be upregulated in BC through circRNA sequencing [13], but its clinical value, biological functions and mechanisms in BC were unknown yet.

In the present study, we further confirmed that circSEMA5A was upregulated in BC tissues and cell lines by qRT-PCR. The overexpressed circSEMA5A was correlated with malignant characteristics of BC. Indeed, many circRNAs showed close association with clinical data of BC patients. For example, lower circMTO1 levels in BC were positively correlated with metastasis and poorer survival of BC patients [19]. Hsa_circ_0000285 was proved to significantly reduced in BC tissues and serum, and associated with cisplatin-resistant, tumor size, differentiation, lymph node metastasis, distant metastasis and TNM stage [20]. Hence, circRNAs might be potential biomarkers for BC.

Functional research suggested that circSEMA5A promoted proliferation, suppressed apoptosis, facilitated migration, accelerated invasion and promoted angiogenesis of BC in vitro. The glycolysis level of BC cells was also raised by circSEMA5A. Tumor cells usually exhibit high-level glycolysis and depend largely on aerobic glycolysis for energy production which is called Warburg effect [21]. The glycolysis not only supplies cancer cell with ATP for cellular energy, but also produce important metabolic intermediates which performs crucial roles in promoting cell proliferation, migration, invasion and evading apoptosis [22, 23]. Taken together, our study confirmed that circSEMA5A is an oncogene in BC and promote malignant processes of BC.

For circRNAs could play a role in a competing endogenous RNAs (ceRNA) manner by acting as miRNA sponges to regulate the expression of miRNA target genes [8, 9]. Here, we demonstrated that circSEMA5A served as a miRNA sponge for miR-330-5p to upregulates ENO1 expression and facilitated the activation of Akt and β-catenin signaling pathways. MiR-330-5p has been confirmed to be a tumor-suppressor gene in many cancers including papillary thyroid cancer [24], glioma [25], oesophageal adenocarcinoma [26] and melanoma [27]. In this study, we found that miR-330-5p was downregulated in BC and functioned as a tumor-suppressor gene in BC. ENO1 has been reported to be a glycolytic enzyme which play vital roles in aerobic glycolysis and Warburg effect in cancer cells [28]. It was proved to act as a key contributor to promotes tumor progression of lung cancer [28], multiple myeloma [29], gastric cancer [30], bladder cancer [31] and so on. What’s more, ENO1 was reported to be able to activate Akt and β-catenin signaling pathways [31, 32], and these two signaling pathways were well-known carcinogenic signaling pathways that closely related to proliferation, apoptosis, migration and invasion. Here, we exhibited that circSEMA5A exerted its biological functions partially via miR-330-5p/ENO1 signaling.

**Figure 8:**
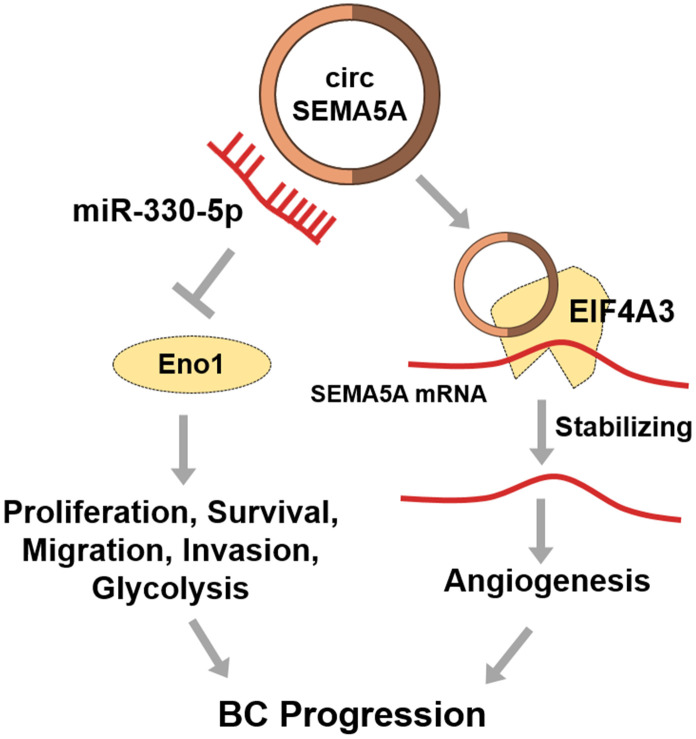
**Diagram showing circSEMA5A regulation pathways in BC.**

Finally, we presented that circSEMA5A could increase SEMA5A expression by recruiting EIF4A3 to enhance the mRNA stability of SEMA5A. EIF4A3 is a RNA binding protein which can be recruited to bind to target RNAs to enhance the RNA stability [33]. SEMA5A has been confirmed to be a transmembrane protein which could be both transmembrane and secreted. SEMA5A can enhance angiogenesis through various ways [14, 34, 35]. Hence, we interpreted that circSEMA5A accelerated BC angiogenesis via regulating SEMA5A. To sum up, in BC, circSEMA5A mediates proliferation, apoptosis, migration and invasion via miR-330-5p/ENO1 signaling, and modulates angiogenesis via SEMA5A (Figure 8).

In conclusion, our present study is the first to uncover the upregulation of circSEMA5A in BC, disclosing that circSEMA5A facilitates bladder cancer progression by mediating miR-330-5p/ENO1 signaling and upregulating SEMA5A expression, indicating the potential value of circSEMA5A as biomarkers and therapeutic target for BC.

## MATERIALS AND METHODS

### Tissue specimens and ethic statement

Forty paired BC tumor and normal tissues were obtained from patients who underwent surgery in the Xinxiang Central Hospital and Beijing Friendship Hospital. Histological and pathological diagnosis were confirmed by two pathologists. This research was approved by The Ethics Committee of Xinxiang Central Hospital and Beijing Friendship Hospital. Informed consents were obtained from all patients.

### Cell culture and transfection

Human BC cell lines T24, UM-UC-3, 5637, J82, the normal human uroepithelial cell line SV-HUC-1 and human umbilical vein endothelial cells HUVEC were obtained from the American Type Culture Collection (ATCC, USA). BC cell lines were cultured in RPM-1640 medium (Gibco, USA) supplemented with 10% FBS (Gibco). SV-HUC-1 cells were cultured in F-12K medium (Gibco) supplemented with 10% FBS (Gibco). HUVECs were cultured in endothelial cell medium (ECM) medium (ScienCell Research Laboratories, USA). Cells were cultured in an incubator at 37°C containing 5% CO_2_. MiRNA mimics, miRNA inhibitors, plasmids, siRNAs were transfected into cells by using Lipofectamine3000 (Life Technologies, USA). CircSEMA5A cDNA was inserted into pcDNA3.1 (+) CircRNA Mini vector (Addgene, USA) to obtain circSEMA5A over-expression plasmids. The target sequences of circSEMA5A siRNAs were: siRNA#1, AGGAGCAAGGCACTTTCCACA; siRNA#2, GTTGTAGGAGCAAGGCACTTT; siRNA#3, AGCAAGGCACTTTCCACACAT; ENO1 or EIF4A3 cDNA was inserted into pcDNA3.1 (+) vector to obtain ENO1 or EIF4A3 over-expression plasmids. ENO1 and EIF4A3 siRNAs were obtained from Santa Cruz Biotechnology (USA).

### RNA preparation and qRT-PCR

Total RNA was extracted from tissues and cells by using RNAiso Plus (TaKaRa, Japan). cDNA was synthesized using PrimeScript RT Master Mix (TaKaRa, Japan). RT-PCR was run on a ABI7300 system (Applied Biosystems, USA) using TB Green Premix Ex Taq II (TaKaRa, Japan). β-actin and U6 were selected as internal controls for circSEMA5A and miR-330-5p. The head-to-tail splicing in the PCR products of circSEMA5A was validated by Sanger sequencing.

### Cell proliferation assay

Cell proliferation was assessed with the 5-Ethynyl-2’-deoxyuridine (EdU) assay. The EdU assay was performed using Cell-Light EdU Apollo567 In Vitro Kit (Ribobio, China) according to the manufacturer's protocol. Images were acquired with an Olympus microscope (Olympus, Japan).

### Cell apoptosis assay

Cell apoptosis rates were detected using Annexin V-FITC Apoptosis Detection Kit (Beyotime, China) according to the manufacturer's protocol. The cells stained by Annexin V-FITC and Propidium Iodide were analyzed by flow cytometry.

### Cell migration assay

Cell migration ability was assessed by wound-healing assays. Cells were cultured and transfected in six-well plates before wounds were made by 200 μL pipette tips (0 h). Then, the cells were cultured with serum-free RPM-1640 medium for 24 h. The scratched lines were photographed at 0 h and 24 h to estimate the cell migration ability.

### Cell invasion assay

Cell invasion ability was assessed by transwell assays which were carried out by 8μm chambers (Costar, USA) with Matrigel (BD Science, USA). Cells (5×10^4^) suspended in 200 μl serum-free RPM-1640 medium were added to the upper chambers and RPMI-1640 medium containing 10% FBS was added into the lower chamber. After incubating for 24 h, cells invading through the membrane were fixed with methanol and stained with 0.1% crystal violet.

### Tube formation assay

Angiogenesis capability was assessed by a tube formation assay which was performed using a 96-well plate coated with 50μL Matrigel (BD Biosciences, USA). A total of 2×10^4^ HUVECs were re-suspended in 100μL BC cell-conditioned medium and seeded on the Matrigel-coated well. The plates were then incubated at 37°C in 5% CO2 for 12h. Images were acquired under a microscope (Olympus, Japan).

### Xenograft model

The animal study was approved by the ethics committee of the Xinxiang Central Hospital and Beijing Friendship Hospital. UM-UC-3 cells (1×10^7^) stably transfected with circSEMA5A shRNA or NC vector were subcutaneously injected into five male BALB/c mice (4 weeks). Then, the mice were maintained for four weeks before being sacrificed and the tumor volume were recorded every week. Tumor volume was calculated with the formula length×width^2^×0.5.

### Metabolite analysis

Cell adenosine 5'-triphosphate (ATP) level was determined by Enhanced ATP Assay Kit (Beyotime, China) according to the manufacturer's protocol. Glucose uptake and lactate release were measured by Glucose Uptake Colorimetric Assay Kit (Sigma-Aldrich) and Lactate Assay Kit (Sigma-Aldrich), respectively.

### Western blot assay

Proteins were extracted using RIPA lysis buffer and nuclear proteins were extracted using ProteoExtract^®^ Subcellular Proteome Extraction Kit (Sigma-Aldrich). The protein concentration was determined with a BCA kit (Thermo Scientific, USA). Primary antibodies used in this assay were Enolase 1 (ENO1) (ab155102, Abcam), β-Catenin (#8480, Cell Signaling Technology), Akt (pan) (#4691, Cell Signaling Technology), Phospho-Akt (Ser473) (#4060, Cell Signaling Technology), Phospho-Akt (Thr308) (#13038, Cell Signaling Technology), SEMA5A (ab127002, Abcam) and β-actin (ab8226, Abcam).

### Fluorescence in situ hybridization analysis (FISH)

Alexa Fluor 488-labeled circSEMA5A oligonucleotide probes and Alexa Fluor 555-labeled miR-330-5p oligonucleotide probes were used to conduct FISH assays. T24 cells were incubated with FISH probe at 42 °C overnight and counterstained with DAPI. Images were acquired under a confocal laser microscopy (Olympus).

### Luciferase reporter assay

Wild-type (wt) and mutant-type (mt) sequences containing predicted binding sites between circSEMA5A and miR-330-5p, miR-330-5p and ENO1 3’UTR were cloned into pmirGLO vector (Promega, USA), respectively. Luciferase reporter vectors and miR-330-5p mimics were co-transfected into T24 cells. After 48 h, luciferase activity was measured by a dual-luciferase reporter assay system (Promega, USA).

### RNA pull-down assay

RNA pull-down assay was conducted using a biotin-labeled probe specific to circSEMA5A back-splice sequence and streptavidin magnetic beads (Life Technologies, CA, USA). Briefly, the beads were incubated with the probes to obtain probe-coated beads. T24 cells were lysed and sonicated, and the cell lysates were incubated with probe-coated beads at 4°C overnight. RNAs captured were isolated by Trizol reagent and subjected to qRT-PCR analysis.

### RNA binding protein immunoprecipitation (RIP) assay

According to the manufacturer’s protocol, RIP assay was carried out using Magna RIPTM RNA Binding Protein Immunoprecipitation Kit (Millipore) and EIF4A3 antibody (ab32485, Abcam) or normal IgG. Co-precipitated RNAs were extracted and analyzed by qRT-PCR assays.

### RNA stability analysis

To block transcription, cells were treated with 10 μg/mL actinomycin D (Sigma-Aldrich). Cells were collected at different time points to detect the remaining level of SEMA5A by qRT-PCR. Expression of 18S rRNA was detected at the same time to serve as an internal control.

### Statistical analysis

Results in this paper were statistically analyzed using SPSS 19.0 statistical software. Data were presented as mean ± standard deviation (SD). Student’s t-test and one-way analysis of variance were used to analyze differences among groups. Chi-squared Test was applied to evaluate the correlation between clinicopathological factors and circSEMA5A expression. *P*<0.05 was considered significant.
